# International recommendations for glucose control in adult non diabetic critically ill patients

**DOI:** 10.1186/cc9258

**Published:** 2010-09-14

**Authors:** Carole Ichai, Jean-Charles Preiser, for the Société Française d'Anesthésie-Réanimation (SFAR), Société de Réanimation de langue Française (SRLF) and the Experts group

**Affiliations:** 1 Medical and Surgical Intensive Care Unit, Saint-Roch Hospital, University of Medicine of Nice, 06000 Nice, France; 2 Department of Intensive Care, Erasme University Hospital, 808 route de Lennik, 1070 Brussels, Belgium; 3 SFAR - Société Française d'Anesthésie et de Réanimation, 74 Rue Raynouard, 75016 Paris, France; 4 SRLF - Société de Réanimation de Langue Française, 48 avenue Claude Vellefaux, 75010 Paris, France

## Abstract

**Introduction:**

The purpose of this research is to provide recommendations for the management of glycemic control in critically ill patients.

**Methods:**

Twenty-one experts issued recommendations related to one of the five pre-defined categories (glucose target, hypoglycemia, carbohydrate intake, monitoring of glycemia, algorithms and protocols), that were scored on a scale to obtain a strong or weak agreement. The GRADE (Grade of Recommendation, Assessment, Development and Evaluation) system was used, with a strong recommendation indicating a clear advantage for an intervention and a weak recommendation indicating that the balance between desirable and undesirable effects of an intervention is not clearly defined.

**Results:**

A glucose target of less than 10 mmol/L is strongly suggested, using intravenous insulin following a standard protocol, when spontaneous food intake is not possible. Definition of the severe hypoglycemia threshold of 2.2 mmol/L is recommended, regardless of the clinical signs. A general, unique amount of glucose (enteral/parenteral) to administer for any patient cannot be suggested. Glucose measurements should be performed on arterial rather than venous or capillary samples, using central lab or blood gas analysers rather than point-of-care glucose readers.

**Conclusions:**

Thirty recommendations were obtained with a strong (21) and a weak (9) agreement. Among them, only 15 were graded with a high level of quality of evidence, underlying the necessity to continue clinical studies in order to improve the risk-to-benefit ratio of glucose control.

## Introduction

Critically ill patients in intensive care units (ICUs) develop insulin resistance that is responsible for so-called "stress diabetes" [1-3]. For a long time this was accepted insofar as stress diabetes was seen as an adaptive metabolic response. However, over the last 10 years, there have been changes in clinical practice resulting from a better knowledge of glucose toxicity and from observations on the benefits of glucose control in clinical trials [4]. Since the first trial in Leuven in 2001 [4], a plethora of articles has been published on the subject but these have triggered much controversy and confused the clinician, with the result that clinical practice varies widely. For this reason, the French Society of Anesthesia and Intensive Care (*Société Française d'Anesthésie-Réanimation*, SFAR) and the French-speaking Society for Intensive Care (*Société de Réanimation de Langue Française*, SRLF) decided to develop expert panel consensus recommendations. Published in 2008 [5], these were updated in May 2009 after the publication of the NICE-SUGAR trial [6]. This paper addresses the practical aspects of glucose control in ICUs, the diagnosis and risks of hypoglycemia, and how to monitor glucose levels in ICU patients.

## Materials and methods

A steering committee, comprising a chair, two SFAR members, and two SRLF members, was set up in late 2007. This committee chose the topics to be addressed and nominated the experts in charge of each specific area. The choice of experts was validated by both societies; 21 French, Belgian and Swiss experts, as well as several medical societies with a stake in the chosen topic, accepted to participate in the development of the recommendations. No member of the committee from industry was present at any of the meetings.

**Figure 1:**
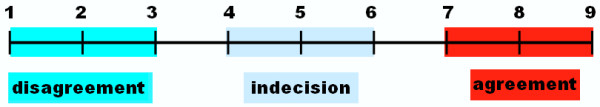
**Process for determination of strong versus weak agreement**. Each expert rated the recommendations on a scale from 1 to 9. A median score ± confidence interval was then calculated based on all expert votes (if necessary, one isolated higher or lower value was excluded). A median score between 1 and 3 indicated disagreement; a median score between 4 and 6 indicated indecision; a median score between 7 and 9 agreement. If the confidence interval was within or without the previous defined zones, the strength of agreement (or disagreement) was considered to be strong or weak, respectively.

The global process for elaborating recommendations is summarised in Additional file 1, Table S1. The aim of the first meeting was to explain the methodology of the working group. Based on a MEDLINE search, each subgroup of experts in charge of its topic had to produce a review including the analysis of the literature and the arguments to propose recommendations. A first version of recommendations was elaborated using the GRADE method (Grade of Recommendation, Assessment, Development and Evaluation) [7,8]. This method takes into account the quality of evidence, the balance between benefits versus harms, endpoint relevance, and costs. As explained during the first meeting, the quality of evidence of each recommendation was systematically specified by the subgroups (Additional file 1, Table S2). The global evidence quality was therefore up- or downgraded by weighting for these three extra factors. Each recommendation was thus allocated a final level of evidence which determined its wording: (i) we recommend (or we do not recommend) for a strong recommendation, (ii) we strongly suggest (or we strongly do not suggest) for a moderate recommendation (iii) we suggest (or we do not suggest) for a weak recommendation (Additional file 1, Table S2). Each recommendation was then rated by all experts on a scale from 1 to 9 (1 = disagreement, 9 = agreement). A median score was calculated (after exclusion of the highest or lowest rating, if necessary) that could fall into one of three zones: (1 to 3) = disagreement; (4 to 6) = indecision; (7 to 9) = agreement. If the confidence interval of the median was within the first or last zone, the strength of the recommendation was considered to be weak or strong, respectively (Figure 1). With this methodology, we must distinguish the strength of recommendation and the level of agreement (or disagreement) obtained from the vote of the experts; for example, it is possible to propose a weak recommendation with a strong agreement. Recommendations for which agreement was not reached in a first round were reworded in order to obtain a better consensus. Up to three rounds were needed to reach an agreement for all recommendations.

Excluding the specific problems of diabetic patients and children, five items were analysed including: i) the glycemic target in ICUs; ii) the diagnosis and consequences of hypoglycemia in ICUs; iii) the rules for carbohydrate intake; iv) the glucose monitoring; and v) the impact of algorithms and protocols. Recommendations are summarized in Additional file 1, Table S3.

## Results

### Glucose target in ICUs

We strongly suggest avoidance of severe hyperglycemia (> 10 mmol/L/180 mg/dL) in adult ICU patients. We suggest keeping glucose levels under control although a universally acceptable upper limit cannot be specified (strong agreement).

We suggest avoidance of tight glucose control in an emergency situation as this management seems to not be reasonable and is potentially dangerous (strong agreement).

We also strongly suggest avoidance of large variations in glucose levels in ICUs (strong agreement).

We do not recommend using any drug other than intravenous insulin for glucose control in ICUs (weak agreement).

### Hypoglycemia: diagnosis and harms

We suggest that in ICU patients, the glucose threshold is probably <2.2 mmol/L (40 mg/dL) for severe hypoglycemia (strong agreement).

In ICU patients unable to express themselves, we recommend that hypoglycemia be corrected even in the absence of clinical signs (strong agreement).

We suggest that severe hypoglycemia is probably associated with an increased risk of mortality although no causal relationship has been established (weak agreement).

Implementation of published strategies for tight glucose control exposes patients to more frequent and long-lasting severe hypoglycemia (strong agreement).

Long-lasting severe hypoglycemia can induce irreversible brain lesions. We suggest that neurological lesions following hypoglycemia might be partly related to excess glucose infusion (strong agreement).

In a strategy of tight glucose control, we recommend closely monitoring glucose blood levels for the early detection of severe hypoglycemia (strong agreement).

We recommend favoring arterial or venous blood samples rather than capillary samples in ICU patients with suspected hypoglycemia as capillary samples often overestimate glucose (strong agreement).

### Carbohydrate intake

We suggest reducing hyperglycemia by restricting intravenous glucose in critically ill patients (weak agreement).

We suggest interrupting intravenous insulin infusion by an electric syringe pump when the patient has resumed food intake and to continue glucose monitoring for at least three preprandial controls (strong agreement).

We cannot suggest a general recommendation of maximal and minimal amounts of intravenous and/or enteral carbohydrates be administered to critically ill patients, regardless of the type, the severity of pathology and of the delay from onset of disease (strong agreement).

We suggest that glucose intake should not be prohibited in critically ill patients provided that glycemia is under control (weak agreement).

We suggest that compliance with the glucose target might be improved by continuous adaptation of enteral nutrition and insulin infusion rates (weak agreement).

### Glucose monitoring

We recommend performing glucose measurements in the laboratory; this remains the current gold standard technique (strong agreement).

We recommend performing glucose measurements in the following preferential order of sampling: arterial, venous, capillary (strong agreement).

As total blood and plasma glucose measurements differ, we recommend knowing the specifications of the device used (not all devices apply an automatic correction factor) (strong agreement).

Owing to endogenous and exogenous physicochemical interference, we recommend being aware of the precise specifications of the device and paper-strips that are used (strong agreement).

### Algorithms and protocols

We recommend defining and implementing a standard protocol for glucose control in each medical team (strong agreement).

Among available glucose control protocols, none may be considered superior to any other (weak agreement).

We recommend including in a glucose control protocol, at the very least, recommendations on the use of rapid action insulin as a continuous infusion by electric syringe pump, as well as on correction and monitoring procedures for episodes of hypoglycemia (strong agreement).

We strongly suggest giving preference to a route of administration providing a constant intravenous insulin infusion rate (strong agreement).

We recommend no longer using static glucose control protocols which determine insulin delivery rate on the basis of the last glucose measurement (strong agreement).

When using glucose control protocols, we strongly suggest taking into account carbohydrate intake in the determination of the insulin delivery rate (strong agreement).

We suggest using a computer-assisted glucose control protocol when there are more than two entries and outputs (weak agreement).

We strongly suggest that the efficacy of a glucose control protocol depends on all of the following criteria: training time, glucose control performance, risk of hypoglycemia, mean error rate, nursing workload (weak agreement).

We suggest assessing the efficacy of a glucose control protocol by considering preferably the following variables: percent time in- and above-target, hyperglycemia index, and variability (weak agreement).

We recommend taking into account the increase in staff workload when implementing a tight glucose control protocol. We recommend allocating time to train the staff before implementing the protocol (strong agreement).

## Discussion

### Glucose target in ICUs

The deleterious impact of hyperglycemia in ICU patients has long been overlooked. However, many observational studies have confirmed that there is a link between hyperglycemia and increased mortality in critically ill patients [9-13]. The decrease in mortality reported in the 2001 Leuven trial after intensive insulin therapy [4] led to a considerable change in clinical practice, with hyperglycemia in ICU patients becoming less acceptable. This trial was a single-center prospective randomized controlled trial (RCT) which compared tight glucose control by intensive insulin therapy (IIT) (4.4 to 6.1 mmol/L) to conventional glucose management (10 to 12.1 mmol/L) in surgical ICU patients. IIT was associated with a decrease in ICU mortality from 8.0 to 4.6% and hospital mortality from 10.9 to 7.2%. The beneficial effects of IIT were greater in patients who spent more than five days in an ICU. A decrease in ICU morbidity was also observed, including lower incidence of systemic infections, acute renal insufficiency, anemia, polyneuropathy, duration of artificial ventilation, and length of stay in the ICU.

However, since the 2001 Leuven trial, the results of several RCTs have dampened the enthusiasm generated by these early results [14-19]. Van den Berghe *et al. *performed the same study in ICU medical patients, with the same objectives and same method, and detected no significant difference in mortality between groups [15]. Two other single-center studies found no decrease in mortality and morbidity in medical and surgical ICU patients receiving IIT [17,18]. Three multicenter RCTs have been performed. The VISEP (Volume substitution and Insulin therapy in severe sepsis) trial assessed the impact of tight glucose control in patients with septic shock or severe sepsis [16]. The 28-day and 90-day mortality rates did not differ between the intensive insulin therapy group (24.7% and 39.7%, respectively) and the conventional treatment group (26% and 35.4%, respectively). Nor did they differ in the GLUCONTROL trial performed in 1,078 medical and surgical ICU patients [19]. The NICE-SUGAR trial in 6,022 ICU patients reported a higher 90-day mortality rate in the tight glucose control group (4.5 to 6 mmol/L) than in the conventional treatment group (< 10 mmol/L) (27.6 vs 24.9%, *P *= 0.02) [14]. Glucose control in ICU patients was found to be beneficial in terms of mortality and morbidity in the oldest meta-analysis [20] but was without effect in the two most recent meta-analyses, even after inclusion of the NICE-SUGAR trial results [21-23].

All these studies are difficult to interpret and to compare because of differences in patient populations and protocol (glucose target levels and measurement methods, carbohydrate intake), and because of methodological weaknesses: single-center studies [4,15,17,18], surgical and/or medical patient populations [4,15,16], early study discontinuation [16,19], and difficulty in reaching the target glucose level [14,16,19]. Currently, it is not possible to establish a universal glucose threshold that might provoke toxicity in ICU patients, irrespective of their disease and environment.

There is no evidence for a benefit of tight glucose control in an emergency situation. Even if hyperglycemia on patient admission to hospital is a marker of a poor prognosis in acute cerebral and cardiovascular disease [24-27], no study so far has shown a short-term benefit of tight glucose control in such emergencies [28-32]. The absence of benefit is largely outweighed by a potentially highly harmful increase in the risk of hypoglycemia.

Several studies have confirmed that acute glucose variations are an independent predictive factor of mortality [13,33-35]. The greater the variations and the closer the mean glucose level to normal, the higher the mortality (the effect is less marked if mean glucose is high >150 mg/dL) [32]. These harmful effects could be related to endothelial dysfunction and increased oxidative stress, although not reported in critically ill patients.

No study has assessed different methods of hyperglycemia management in ICUs. The need for optimal efficacy (reaching target values and minimizing variations) and for maximum safety (reducing the incidence of hypoglycemia) is nevertheless a strong argument in favor of continuous intravenous insulin infusion by an electric syringe pump. In ICU patients with edema or vasomotor variations, intravenous infusion minimized fluctuations in insulin absorption and enabled delivery to be adapted fast and effectively to variations in glucose levels [36,37]. By adjusting insulin delivery rate in advance, it might be possible to prevent hyperglycemia induced by glucose intake (food) or drugs (glucocorticoids), but no study addressed this question in critically ill patients. Subcutaneous insulin absorption is unreliable and may be unpredictable in patients with edema or shock; glucose control occurs haphazardly [38]. In a perioperative study in diabetic patients, target values were reached in only 40% of patients after subcutaneous insulin [36].

### Hypoglycemia: diagnosis and harms

The definitions of hypoglycemia and its severity are well established for diabetic patients [39,40]. A third party has to be present to confirm the degree of severity before oral or intravenous glucose may be administered. However, there are no published data or definitions for hypoglycemia in ICU patients. Unlike in diabetic patients, it is arbitrarily and exclusively defined on the basis of a biological threshold without taking any account of neurologic signs. Most studies conducted in ICUs were not designed to assess hypoglycaemia and rely only on the definition based on the blood glucose concentration, regardless of associated clinical signs (< 2.2 mmol/L) [3,4,14-18,41].

The definition of severe hypoglycemia used in diabetic patients cannot be transposed directly to ICU patients who may be unable to describe clinical signs because of spontaneous or sedation-induced consciousness disorders. Other cardiovascular clinical signs may also escape attention. The lack of a specific sign and the inability to detect early warning signs increase the risk of severe hypoglycemia [3,19,41]. Most cases of hypoglycemia described in ICU trials are of short duration (< 2 hours) and exclusively biology-based with no report of a clinical sign of severity [42].

In most studies, hypoglycemia is associated with a significant increase in mortality (relative risk: 2.3 to 3.8) [4,16,19,43,44]. Other studies have, however, suggested that hyperglycemia is not an independent predictive factor of mortality [45-47]. Current evidence can therefore neither refute nor establish a causal relationship. Recent data have, however, highlighted factors that predispose to hypoglycaemia such as continuous haemofiltration, diabetes, mechanical ventilation, sepsis, administration of insulin and inotropic drugs [45-47], and brain lesions [48]. In such situations, the effects of a strategy targetting a higher glucose target level should be evaluated.

Most ICU studies use *at least one *episode of severe hypoglycemia as a yardstick to report hypoglycemia incidence. The incidence (5 to 25% according to study) is always significantly higher than in the control group. The most recent studies report a three- to six-fold increased risk of severe hypoglycemia [20,22,45-55].

The available evidence related to the clinical consequences of long-lasting severe hypoglycemia and its correction is not reported from critically ill patients. In experimental models, post-hypoglycemic neuronal death is not directly due to an energy deficit but arises from a cascade of reactions triggered by hypoglycemia, in particular a glutamate and zinc influx that activates post-synaptic glutamate receptors. This leads to numerous cellular modifications (for example, production of reactive oxygen species (ROS), DNA modifications and impairment of membrane permeability) resulting in neuronal apoptosis [49]. Using an experimental model for severe hypoglycemia, Suh *et al. *showed that neuronal death hardly occurred during hypoglycemia but was marked during glucose reperfusion [50]. Neuronal death was proportional to the hyperglycemic rebound induced by exogenous glucose reperfusion, and was induced by NADPH oxidase, responsible for ROS production. This is reminiscent of the mechanisms of cellular death during episodes of reperfusion following ischemia. Despite the lack of clinical evidence supporting these experimental data, and because of variability in glucose levels, more rigorous management of hypoglycemia (infusion of a more moderate amount of glucose and closer monitoring) could be needed to prevent an excessive hyperglycemic rebound.

The higher incidence of hypoglycemia during tight glucose control, associated with the frequent absence of clinical warning signs, calls for repeated glucose measurements. However, there is no study that can be used as a basis to recommend any given interval between measurements as a function of the equilibrium observed: from 30 minutes (in cases of hypoglycemia or severe hyperglycemia) to 4 hours depending upon glucose level stability and study [4,15-19].

Irrespective of measurement method, glucose levels vary according to sampling site, as recently confirmed in patients with shock or edema [51-55]. Values measured on capillary samples are overestimated compared to those measured on arterial samples [53,54]. The discrepancy would be 30% according to the most recent data [14,53]. However, approximate measurements for non severe hypoglycemia are not acceptable in patients with no clinical signs of severity. A control measurement should be performed on arterial or venous blood in the laboratory or using a blood gas/glucose analyzer. This approach, widely used in diabetics [40], was applied in the recent NICE-SUGAR trial [14]. There have been reports of episodes of severe hypoglycemia that have remained undetected by point-of-care capillary blood analyzers [56].

### Carbohydrate intake

Hyperglycemia probably has beneficial or harmful effects depending upon the mechanism of its onset, its severity, and duration [41]. Stress diabetes is a transitory abnormality induced by acute disease (inflammation, ischemia-reperfusion) and a marker of disease severity. It is also an adaptive response for overcoming the acute metabolic changes observed in ICU patients [3,57-59]. Faster glucose turnover and insulin resistance initially provide the amount of energy substrate (glucose) that some organs need [57,60,61]. Hypoxia and proinflammatory phenomena (cytokines) intensify this *endogenous *hyperglycemia, and *vice-versa*, thus creating a vicious circle. The hyperglycemia can be worsened and prolonged by the development of *exogenous *hyperglycemia through enteral or parenteral glucose intake or glucocorticoid administration. The glucose that was initially useful is now present in excess and becomes toxic by enhancing inflammatory responses and inducing oxidative stress [62-64]. The different outcomes in the Leuven and NICE-SUGAR trials might be partly due to differences in carbohydrate intake levels. Van den Berghe *et al. *administered high carbohydrate levels (200 g/day) [4]. This could have enhanced glucose toxicity. The glucotoxicity would have been reversed by intensive insulin therapy. In contrast, enteral carbohydrate administration in the NICE-SUGAR trial was restricted especially during the first two to three days [14]. Early insulin administration to induce a return to normal glucose values might have worsened the patients' conditions by preventing an adaptive response.

There is no evidence justifying either the continuation or interruption of intravenous insulin therapy once ICU patients have resumed food intake. The duration of glucose monitoring in ICUs has not been investigated in a well-designed study (except in diabetic patients). According to physiopathological data, it is reasonable to expect that patients who can eat have recovered glucose regulation with appropriate endogenous insulin secretion, in particular before meals. All RCTs have used the following regimen: intravenous or subcutaneous preprandial insulin bolus with at least one glucose measurement before each meal [4,14,19]. Glucose monitoring was stopped once the patient left the ICU. Some studies have recommended substituting subcutaneous for intravenous insulin before the patient leaves the ICU [65]. A retrospective study in neurosurgery patients has shown that 6 to 70% of the intravenous insulin dose, administered by the subcutaneous route, provided satisfactory glucose control with no increase in risk of hypoglycemia [66].

The recommended daily energy intake in ICU patients is about 25 kcal/kg/day [67]. It may take at least two to three days to reach this objective. If the enteral calorie intake is still too low after three days, parenteral supplementation may be used [67]. Glucose is a key energy substrate; some tissues depend totally or highly on glucose. Mean daily consumption by the brain is 100 to 150 g. The source may be exogenous or endogenous. Exogenous glucose comes from enteral or parenteral carbohydrate intake. Endogenous glucose comes mostly from hepatic or muscular neoglucogenesis and can reach 300 g/day [68]. ICU patients are insulin resistant and too much exogenous glucose increases the risk of hyperglycemia [1], in particular as maximum glucose oxidation capacity is reduced to 2 to 5 mg/kg/minute [57,69,70]. In such a situation, glucose infusion only partially inhibits neoglucogenesis. However, these observations apply to short periods (less than three days) in cohorts of critically ill ICU patients [71], and assessment of the impact of enteral carbohydrates on glucose metabolism remains difficult (the estimated true digestive absorption is not very reliable). On the other hand, no, or very little, exogenous glucose may hasten neoglucogenesis substrate use and muscle protein catabolism. In summary, total glucose deprivation (fasting) or a too high intake clearly have harmful effects in ICU patients. However, optimal carbohydrate intake has still to be established [67].

The impact of carbohydrate intake on glucose levels in ICU patients suggests that glucose control protocols should take account of carbohydrate intake [72]. In theory, this should achieve optimal glucose control by foreseeing variations in glucose levels (hyper- and hypoglycemia). According to several reports, the performance of glucose control software accounting for carbohydrate intake is satisfactory [73-77]. However, its benefits have yet to be demonstrated in routine clinical practice.

### Glucose monitoring

The gold standard measurement is one made in the laboratory on an arterial or venous blood sample using hexokinase [78,79]. Point-of-care glucometers use other enzymes (glucose oxidase (GO) or glucose dehydrogenase (GDH)). GO is the enzyme used in the older models. It is less stable than GDH and therefore less precise, and has more limitations. Point-of-care glucose readers must comply with strict standards (ISO 15197 in Europe) regardless of the enzyme used, that is, a deviation with respect to the gold standard of <15 mg/dL for glucose levels above 75 mg/dL and a maximum 20% deviation for higher levels [80]. Most devices meet these standards but none yields a more accurate measurement (< 10% deviation) [52,53].

The sampling site may influence glucose measurements and be a source of discordant values. Glucometers may well comply with international standards, but they were devised to measure glucose in capillary blood from ambulatory patients. The reliability of their use in ICU patients is a matter of controversy [51,52,54,55,80]. The main sources of discrepancies are vasoconstriction, low blood flow rate, a state of shock, ischemia, or edema [54,78,79]. In such cases, about 15% of capillary measurements vary by >20% with respect to the gold standard [78,81]. The discrepancies are worse in cases of hypoglycemia, thus justifying confirmation in the laboratory [54]. Measurements on arterial blood show the least variation.

As plasma is richer in water than red blood cells, glucose measurements on plasma are higher than on total blood, by about 10 to 15% [79]. The discrepancy is even greater in cases of abnormal hematocrit values. The World Health Organisation (WHO) recommends that plasma values be converted into laboratory total blood values by applying a correction factor of 1.12. However, plasma glucose does not depend on the hematocrit value and reflects active glucose more faithfully. For this reason, and in order to avoid any errors in interpretation, the American Diabetes Association and the International Federation of Clinical Chemistry and Laboratory Medicine Scientific Division (IFCC) have urged that practice be harmonized by considering plasma glucose only, regardless of sampling site and measuring device [79]. They recommend a correction factor of 1.1 to be applied to results for total blood. Most recent devices using paper-strip blood sampling have in-built automatic correction and provide plasma values [80,82].

Point-of-care glucose meters use different measurement methods (amperometric or colorimetric reaction, enzymatic reaction (GO or GDH), calibration on total blood or plasma, and different blood volumes) which all lead to device-specific limitations, interferences, and technical constraints [82-86]. GO systems (the oldest) are influenced by blood oxygen concentration as oxygen is involved in catalysis. GDH devices use either PQQ (pyrroloquinolone quinone) or FAD (flavine adenine dinucleotide) for catalysis. Depending upon the device, certain physicochemical factors can impair measurement accuracy. Sampling conditions and interpretation of results must therefore take the type of device into account [78]. The PaO_2 _value, very high or low pH values, hypothermia, and altitude can influence measurements made with GO devices [87,88]. With the oldest GO devices (amperometric reaction), PaO_2 _values of <40 mmHg may overestimate glucose by about 15%. The more recent GO devices (colorimetric reaction) are more reliable over a wide PaO_2 _range [87]. GDH devices are not affected by the PaO_2 _value, but high galactose or maltose concentrations may overestimate values given by GDH PQQ devices. Cases of wrong results resulting in the death of the patient have led to banning their use in such situations [89,90]. All GDH devices (PQQ or FAD) overestimate values in the presence of high concentrations of some substances (endogenous substances: uric acid, bilirubin, triglycerides; exogenous substances: xylose, salicylate, paracetamol, mannitol). This information is supplied in the manufacturer's instructions [78]. Many continuous glucose monitoring systems (subcutaneous or intravascular measurements) are being developed and assessed. Their reliability in ICUs has yet to be demonstrated [3]. In summary, the reliability of the results depends on the user's knowledge of the device.

### Algorithms and protocols

The early results of Van den Berghe *et al. *led to the widespread use of continuous insulin therapy for glucose control. Hospital teams drafted protocols to promote efficacy and safety. A wide variety of algorithms have been published because the choice of criteria is vast: target glucose, insulin delivery rate, monitoring interval, management by doctors or nurses, and so on. In Van den Berghe *et al.*'s trial, the algorithm was implemented by a specially trained nursing staff [4]. On the other hand, in the NICE-SUGAR trial, a web-based computerised protocol with several entries was used to provide insulin delivery rates and monitoring intervals [14]. In all cases, a written protocol suited to local conditions (technical and human resources) and accepted by the care team should be implemented in order to guarantee efficacy and safety [91-93].

No prospective RCT has compared the impact of glucose control protocols on morbidity and mortality. It is difficult to assess algorithm performance because of the variety of variables used. Currently, there is no evidence for choosing one protocol rather than another.

Continuous intravenous insulin provides greater efficacy, safety, and ease of use than subcutaneous administration in ICUs [3,41,91,94,95]. It is used by virtually all ICUs and is sometimes supplemented by intravenous boli. It has the advantage of limiting wide variations in glucose; this is as important as the mean hyperglycemia value [13,12,34,41]. In addition, although a causal relationship between hypoglycemia and increased mortality has not been proven, it is prudent to recommend glucose control techniques that limit these episodes as far as possible [65].

A study of 100 ICU patients has shown that the incidence of severe hypoglycemia was significantly reduced when insulin was administered by a specific rather than non specific infusion route (4% vs 22%) [96]. As for continuous catecholamine administration, this helps avoid any variations in delivery that may be induced by the injection of other drugs.

Static control algorithms determine insulin delivery rate from a single (the last) glucose measurement. Dynamic control algorithms take a wide variety of other factors into account such as the ongoing insulin delivery rate, monitoring interval, glucose intake, and so on. This accounts for protocol diversity. Available evidence shows that dynamic control is better than static control [91]. The approach used should also take account of exogenous glucose intake which may affect glucose levels [72-77]. Ideally, intake should be anticipated in order to achieve more stable glucose levels [3].

Entry variables are those that spark off recommendations whereas output variables are those that make up the recommendations. The entry invariably used is glucose value but other entries such as previous insulin delivery rate and the monitoring interval may be taken into account. The output common to all algorithms is the insulin delivery rate. Other possible outputs are recommendations concerning insulin boli, food intake, monitoring interval, hypoglycemia correction, and so on. The number of entries and outputs make non computer-assisted protocol management well-nigh impossible. The complexity of the paperwork of the NICE-SUGAR trial might explain the limited time spent in-target (40%), the low proportion of eligible patients (15%), and the short monitoring intervals increasing workload [14]. Dedicated computer software is being developed [76,77,97-99]. There are two types of computer-assisted second generation software using complex algorithms: (i) Proportional-Integral-Derivative (PID) software uses a closed-loop control that takes into account the deviations with respect to target glucose value, time in-target, and variations in level [77,100]. The changes in insulin delivery rate are always based on past measurements; (ii) Model Predictive Control (MPC) software predicts glucose values using established models [74,76,98].

An effective glucose control protocol does not only consider attainment of the target glucose value but also protocol adoption time by staff, risk of hypoglycemia, and the variability and reliability of measurements [73-77,91].

The efficacy of glucose control depends on factors that differ considerably among studies. Recent work has tried to establish the factors needed to validate protocol efficacy [101-103]. The most important seem to be hyperglycemia index and variability. The frequency and severity of hypoglycemia reflect protocol safety.

The introduction of glucose control in ICUs increases staff workload because of protocol implementation time and repeated monitoring. In a prospective single-center study, the time required was two hours per day, that is, about 20% of a nurse's working day [104]. For a protocol to be effective and safe, its feasibility should be tailored to resources; close cooperation is needed between doctors and nurses for the procedure to take account of local technical and human resources. Users must accept the protocol and training [105]. Despite these measures, failure in reaching the target glucose value has been reported in over 30% of patients [106].

## Conclusions

Glucose control in ICUs should be a therapeutic objective. It is no longer possible to overlook severe hyperglycemia (> 10 mmol/L) although it is not yet possible to recommend a single glucose threshold common to all types of patients and diseases, especially as glucose control exposes patients to an increased risk of potentially harmful hypoglycemia. In addition, although mean glucose is an important therapeutic target to be achieved, recent data underscore the impact of many other factors (for example, variability in glucose levels, carbohydrate intake, presence or not of chronic hyperglycemia (diabetes). The safety and reliability of glucose monitoring techniques also need to be taken into account. Progress in the accuracy, harmonisation, and automation of these techniques is needed to enhance the efficacy and safety of glucose control, and diminish workload. There is no question of introducing tight glucose control into ICUs at all costs. However, further studies are needed to answer many unsolved questions: Which target glucose values should be used in which patients? How to monitor glucose levels? Which protocols should be used? In the meantime, each team should set up formal protocols in line with their technical and human resources.

## Key messages

• Stress-induced hyperglycemia has been found to be associated with an increased morbi-mortality in critically ill patients. Thus, an excessive hyperglycemia (> 10 mmol/L) should be avoided in adult ICU patients.

• Due to persistent conflicting data and the increased risk of hypoglycemia, strict glycemic control cannot be a universal strategy regardless of the condition of patient and the training of the team.

• Continuous intravenous insulin is the only strategy permitted to efficiently control glycemia while decreasing the risk of glycemic variations in critically ill patients.

• In ICU, severe hypoglycemia (< 2.2 mmol/L) should be detected, even in the absence of warning clinical signs, using a close glycemic monitoring (repeated blood samples).

• Blood glucose concentrations determined with bedside point-of-care glucometers provides inaccurate measurements in critically ill patients. Thus, blood glucose measures should be preferentially performed on arterial (or venous) blood samples using classical laboratory devices or blood gas/glucose analyzers, especially in the case of extreme values.

## Abbreviations

FAD: flavine adenine dinucleotide; GDH: glucose dehydrogenase; GO: glucose oxidase; GRADE: Grades of Recommendations, Assessment, Development And Evaluation; ICUs: intensive care units; IFCC: International Federation of Clinical Chemistry and Laboratory Medicine Scientific Division; IIT: intensive insulin therapy; MPC: Model Predictive Control; PID: Proportional-Integral-Derivative; PQQ: pyrroloquinolone quinone; RCTs: Randomized Control Trials; ROS: Reactive oxygen species; VISEP: volume substitution and insulin therapy in severe sepsis; WHO: The World Health Organisation.

## Competing interests

PK (Experts Group) is a shareholder of LK2 society, 37554 Saint Avertin, France. All other authors declare that they have no competing interests.

## Authors' contributions

CI initiated the study, proposed to the SFAR and SRLF to support it and organized and supervised the meetings and the experts. All members of the Experts Group were responsible for the analysis of the literature, the elaboration of the recommendations related to their topic and the validation of the whole final recommendations. The Steering Committee was responsible for control of the method and the final elaboration of recommendations. CI and JCP wrote and drafted the final manuscript. All authors read and approved the final manuscript.

## Supplementary Material

Additional file 1 **Tables S1, S2 and S3**. **Table S1**. Successive process for developing recommendations; **Table S2**. Grading quality of evidence and strength of recommendation; **Table S3**. Experts recommendations for glucose control in ICU. Click here for file
