# Supplementary material for: International recommendations for glucose control in adult non diabetic critically ill patients
Source: Crit Care. 2010 Sep 14;14(5):R166. doi: 10.1186/cc9258 (PMC3219261; doi:10.1186/cc9258)
Supplement: Additional file 1 — Tables S1, S2 and S3. Table S1. Successive process for developing recommendations; Table S2. Grading quality of evidence and strength of recommendation; Table S3. Experts recommendations for glucose control in ICU. [file cc9258-S1.DOC]

**Additional file 1**

**Table S1.** Successive process for developing recommendations

| **1. The Steering Committee**  - Is designated by the Société Française d'Anesthésie-Réanimation (SFAR) and the Société de Réanimation de Langue Française (SRLF)  - Is responsible of the selection of the topics and the experts  - Validation by both French Societies SFAR and SRLF |
| --- |
| **2. First meeting = description of the methodology**  - Distribution of the appropiate topic to each subgroup of experts  - Role of each subgroups of experts:  . to provide a manuscript including the analysis of the lottaretue (MEDLINE search)  . give the arguments issued from the manuscript leading to formalize the recommendations with their level of evidence (GRADE method) |
| **3. Subsequent meetings**  - All subgroup of experts provided their manuscript and recommendations  - Determination of a consensual final level of evidence for each recommendation leading to formulate : strong, moderate or low strength of recommendation  - Determination of the level of agreement (or disagreement) of the experts by a cotation based on a scale from 1 (strong disagreement) to 9 (strong agreement) (see figure 1)  - Reconsideration of recommendation with no agreement up to 3 tours to obtain the agreement |

**Table S2.** Grading quality of evidence and strength of recommendation

| **Criteria for evidence**  - Type of evidence:  . randomized trial = high  . observational study = low  . any other study = very low  - Decrease or increase grade according to:  . endpoint relevance  . balance benefits and harms  . costs |
| --- |
| **Strength of recommendation**  - Strong level of evidence = one or more meta-analysis in agreement with high evidence; one or 2 trials in agreement with high evidence: we recommend (or we do not recommend)  - Moderate level of evidence = 2 trials in agreement with low evidence: we strongly suggest (or we do not strongly suggest)  - Low level of evidence = several contradictory trials with low evidence: we suggest (or we do not suggest) |

**Table S3. Experts recommendations for glucose control in ICU**

| **Recommendations** | **Strength of agreement** |
| --- | --- |
| **Glucose target in ICUs**  . We **strongly suggest** to avoid severe hyperglycemia (> 10 mmol/L - 180 mg/dL) in adult ICU patients. We **suggest** to keep glucose levels under control although a universally acceptable upper limit cannot be specified.  . We **suggest** to avoid tight glucose control in an emergency situation as this management seems to be not reasonable and potentially dangerous.  . We **strongly suggest** to avoid large variations in glucose levels in ICUs.  . We **do not recommend** to use any drug other than intravenous insulin for glucose control in ICUs. | Strong agreement  Strong agreement  Strong agreement  Weak agreement |
| **Hypoglycemia: diagnosis and harms**  . We **suggest** that in ICU patients, the glucose threshold is probably < 2.2 mmol/L (40 mg/dL) for severe hypoglycemia.  . In ICU patients unable to express themselves, we **recommend** that hypoglycemia be corrected even in the absence of clinical signs.  . We **suggest** that severe hypoglycemia is probably associated with an increased risk of mortality although no causal relationship has been established.  . Implementation of published strategies for tight glucose control **exposes** patients to more frequent and long-lasting severe hypoglycemia.  . Long-lasting severe hypoglycemia **can induce** irreversible brain lesions. We **suggest** that neurological lesions following hypoglycemia might be partly related to excess glucose infusion.  . In a strategy of tight glucose control, we **recommend** to monitor closely glucose blood levels for the early detection of severe hypoglycemia.  . We **recommend** to favor arterial or venous blood samples rather than capillary samples in ICU patients with suspected hypoglycemia as capillary samples often overestimate glucose. | Strong agreement  Strong agreement  Weak agreement  Strong agreement  Strong agreement  Strong agreement  Strong agreement |
| **Carbohydrate intake**  . We **suggest** to reduce hyperglycemia by restricting intravenous glucose in critically ill patients.  . We **suggest** to interrupt intravenous insulin infusion by electric syringe pump when the patient has resumed food intake and to continue glucose monitoring for at least 3 preprandial controls.  . We **cannot suggest** a general recommendation of maximal and minimal amounts of intravenous and/or enteral carbohydrates to be administered to critically ill patients, regardless of the type, the severity of pathology and the delay from onset of disease.  . We **suggest** that glucose intake should not be prohibited in critically ill patients provided that glycemia is under control.  . We **suggest** that compliance with the glucose target might be improved by continuous adaptation of enteral nutrition and insulin infusion rates. | Weak agreement  Strong agreement  Strong agreement  Weak agreement  Weak agreement |
| **Glucose monitoring**  . We **recommend** to perform glucose measurements in the laboratory which remains the current gold standard technique  . We **recommend** to perform glucose measurements in the following preferential order of sampling: arterial, venous, capillary.  . As total blood and plasma glucose measurements differ, we **recommend** to know the specifications of the device used (not all devices apply an automatic correction factor).  . Owing to endogenous and exogenous physicochemical interference, we **recommend** to be aware of the precise specifications of the device and paper-strips that are used. | Strong agreement  Strong agreement  Strong agreement  Strong agreement |
| **Algorithms and Protocols**  . We **recommend** to define and implement a standard protocol for glucose control in each medical team.  . Among available glucose control protocols, none **may be considered** superior to any other.  . We **recommend** to include in a glucose control protocol, at the very least, recommendations on the use of rapid action insulin as a continuous infusion by electric syringe pump, as well as on correction and monitoring procedures for episodes of hypoglycemia.  . We **strongly suggest** to give preference to a route of administration providing a constant intravenous insulin infusion rate.  . We **recommend** to no longer use static glucose control protocols which determine insulin delivery rate on the basis of the last glucose measurement.  . Using glucose control protocols, we **strongly suggest** to take into account carbohydrate intake in the determination of insulin delivery rate.  . We **suggest** to use a computer-assisted glucose control protocol when there are more than 2 entries and outputs.  . We **strongly suggest** that the efficacy of a glucose control protocol depends on all of the following criteria: training time, glucose control performance, risk of hypoglycemia, mean error rate, nursing workload.  . We **suggest** to assess the efficacy of a glucose control protocol by considering preferably the following variables: percent time in- and above target, hyperglycemia index, and variability.  . We **recommend** to take into account the increase in staff workload when implementing a tight glucose control protocol. We **recommend** to allocate time to staff training before implementing the protocol. | Strong agreement  Weak agreement  Strong agreement  Strong agreement  Strong agreement  Strong agreement  Weak agreement  Weak agreement  Weak agreement  Strong agreement |
